# Microstructure and Mechanical Properties of Tungsten Inert Gas Weld Joints of Sprayed and Cast Aluminium–Lithium Alloy

**DOI:** 10.3390/ma13173787

**Published:** 2020-08-27

**Authors:** Chuanguang Luo, Huan Li, Yuhui Zhang, Jianguo Li, Yuanhua Wen, Lijun Yang

**Affiliations:** 1 Tianjin Key Laboratory of Advanced Joining Technology, Tianjin University, Tianjin 300072, China; chg_luo@163.com (C.L.); lihuan@tju.edu.cn (H.L.); yuhui9256@163.com (Y.Z.); jgli1991@163.com (J.L.); 2 Sichuan Aerospace Changzheng Equipment Manufacturing Co., Ltd., Chengdu 610100, China; wyh20017@163.com

**Keywords:** aluminium–lithium alloy, Tungsten Inert Gas Weld, microstructure, tensile strength, elongation

## Abstract

The weld joints of sprayed 2195-T6 and cast 2195-T8 aluminium–lithium alloy were created using tungsten inert gas with filler wire. The microstructures and mechanical properties of the weld joints were examined. The results of the microstructure analysis showed that the width of the equiaxed grain zone (EQZ) and the amount of the second phase θ’(Al_2_Cu) was greater in the weld joint of the cast 2195-T8 Al–Li alloy than that of the sprayed 2195-T6 Al–Li alloy. Tensile testing indicated that failures occurred in the EQZ and partially melted zone (PMZ) for both weld joints. The tensile strength and elongation of the weld joints of the sprayed 2195-T6 and cast 2195-T8 Al–Li alloys were about 68.2%, 89.7%, and 50.7% and 28.3% those of the base metal in the joint, respectively. The cast 2195-T8 Al–Li alloy joint had more pores and cracks, resulting in lower tensile strength and elongation than those in the sprayed alloy. Further, the tensile fracture surface morphology indicated that the fracture mode of the sprayed 2195-T6 Al–Li alloy was a mixed fracture mode dominated by plastic fracture and that of the cast 2195-T8 Al–Li alloy joints was a mixed fracture mode dominated by brittle fracture.

## 1. Introduction

With the development of the aerospace industry, the need for lightweight materials with strong mechanical properties is increasing. Al–Li alloys are widely used in the aerospace industry owing to their low density, high strength, and other excellent mechanical properties. El-Aty et al. [1] described the history of Al–Li alloys and the superior performance of third-generation Al–Li alloys, which include 2195 Al–Li alloys. By adjusting the ratio of Cu to Li in the alloy, a large number of finely dispersed T_1_(Al_2_CuLi) phases can be obtained, and the strength of the aluminium alloy has been improved [2]. The strength and ductility of Al–Cu–Li alloys can be increased through a series of solid–solution, deformation, and ageing treatment methods. According to previous studies, the performance of cast Al–Li alloy has been significantly improved [3,4]. However, further improvements in performance have been limited as the alloy has larger dendritic and elements of Zn, Cu, and Mg segregation in a cast state [5,6]. A sprayed alloy was obtained using a superheated liquid alloy and then atomised into a fine dispersion of 5–40 μm droplets using nitrogen gas. The small droplets fall onto a rotating substrate, forming a billet of sprayed alloy. The alloy spray combines the excellent characteristics of powder metallurgy and casting, with fine crystal grains and a small macrosegregation, allowing precise control of the alloy composition. The alloy spraying technique has been successfully applied to seven series aluminium alloys [7,8]. 

There are many welding methods used to join Al–Li alloys, such as friction stir welding (FSW), electron beam welding (EBW), laser beam welding (LBW), and tungsten inert gas (TIG). In [9], the authors showed that the ultimate tensile strength and elongation only reach 65% and 54% of the base metal when FSW was used to cast 2195-T8 aluminium alloy. The EBW of Al–Cu–Li alloy was also studied [10]; the results showed that the weld joint achieves a porosity of only 0.04% and leads to a tensile strength of 68% of the base metal, along with a lower elongation when the heat input is approximately 64.8–94.8 J/mm. In [11], the authors investigated the LBW of AA2060 Al–Li alloy with an AlSi12 filler wire with a joint tensile strength of 83% of the base metal when the heat input was approximately 80 J/mm. Although FSW, EBW, or LBW can be used to increase the joint strength of Al–Li alloys after welding because of the lower heat input, FSW, EBW and LBW are inconvenient for the welding of larger engineering components owing to the need for auxiliary equipment. For example, the manufacture of a large-diameter rocket tank needs professional fixture and special welding equipment. In addition, a T-shaped flange is unsuitable for FSW. By contrast, the TIG welding method requires less equipment and is more flexible and convenient. Solórzano et al. [12] stated that the ultimate tensile strength of the joint in a TIG-welded Al–Li alloy 2091 without filler wire was approximately 63% of the base metal. In addition, Chen and Chaturvedi [13] proposed a double-V-groove of TIG-welded cast Al–Li alloy 2195 using a 4043 Al–Si filler wire and showed that the ultimate tensile strength and ductility of the joint were approximately 51.6% and 16.4% those of the base metal, respectively. Despite the convenience of the TIG method, the mechanical properties of the weld joints obtained are lower than the mechanical properties obtained using other methods, such as FSW, and, thus, the method requires further improvement.

Although previous studies on the TIG welds of Al–Li alloys have been conducted, the differences and relationship between sprayed and cast 2195 Al–Li alloy joints have not been investigated. This study aims to determine the mechanical properties of sprayed 2195-T6 and cast 2195-T8 Al–Li alloy joints using TIG welding. The differences in the microstructure and mechanical properties between the two joints were analysed using electron microscopy and a tensile machine. The width of the equiaxed grain zone (EQZ), the distribution of Cu, and the microhardness, tensile properties, and fracture mode of the joints were investigated.

## 2. Materials and Methods

### 2.1. Materials

Aluminium–lithium alloy 2195 was used in this study. Prior to the welding process, the materials were placed under T6 and T8 conditions. The chemical compositions of the base metal and filler wire are listed in Table 1. The cast 2195-T8 alloy was supplied by Alcoa(Aluminium company of America, Pittsburgh, USA) and the sprayed 2195-T6 alloy was supplied by Jiangsu Haoran Spray Forming Alloy Co., Ltd, Zhenjiang, China. Both materials met the Unified Numbering System for Metas and Alloys (UNS) A92195 chemistry requirements, as shown in Table 1. Qin et al. [14] showed that 2195 Al–Li alloy is a precipitation-strengthened alloy with the main strengthening phase of T_1_(Al_2_CuLi). Other elements, such as Li, Zr, and Ti, refine the microstructure and effectively promote heterogeneous nucleation. Other phases such as θ’(Al_2_Cu), δ’(Al_3_Li), and β’(Al_3_Zr) also have certain effects on the strengthening of the alloy. The mechanical properties of the base materials are listed in Table 2.

**Figure 1:**
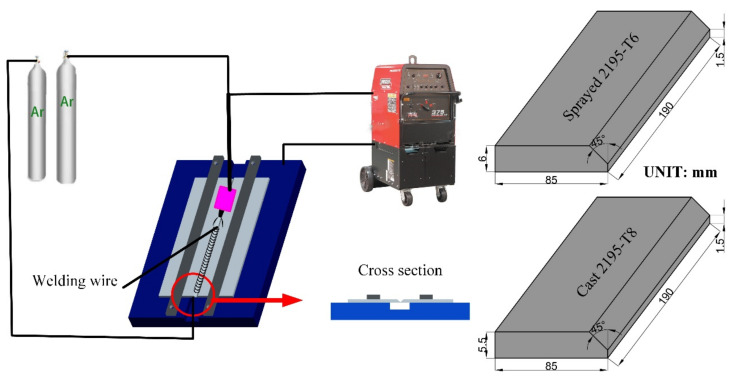
Schematic of the experimental system and 3D dimensions of base metal.

The plate specimen geometry prior to welding is shown in Figure 1. The specimen with sprayed 2195-T6 alloy has a thickness of 6.0 mm and the specimen with cast 2195-T8 alloy has a thickness of 5.5 mm. A slope of 45° along with an edge thickness of 1.5 mm was prepared on one side of the plate prior to the welding. The filler wire was 3.2 mm in diameter. Prior to the welding, the surface of the filler wire was cleaned with a spatula and then wiped with acetone. The plate specimen was first mechanically polished with a brush and then cleaned with acetone. The plate samples were then used within 2 h of cleaning.

### 2.2. Welding Equipment

The experimental setup is shown in Figure 1. The welding process was applied using a Lincoln welder (Lincoln Precision TIG 375, Lincoln Electric, Cleveland, OH, USA) with an AC square wave and argon gas with a purity of 99.999%. The argon gas was provided to the torch and the back of the weld seam simultaneously. The flow rate of the argon gas to the torch was 14 L/min, and the flow rate to the back of the weld seam was 13 L/min. The ambient temperature and humidity were 29 °C and 42%, respectively. The welding parameters are listed in Table 3.

### 2.3. Microstructure and Microhardness Test

The transverse cross-sections of the welded joints were embedded in an epoxy resin, polished, and etched using Keller’s reagent (5 mL of HNO_3_, 3 mL of HCL, 2 mL of HF, and 190 mL of H_2_O) for 18 s. The morphology of the joint was observed using an optical microscope (OM) (Zeiss Vert. A1, Carl Zeiss AG, Oberkochen, Germany) and a Zeiss Smartzoom 5 microscope (Carl Zeiss AG, Oberkochen, Germany) with an ultrawide depth of field. In addition, SU-1510 and JSM-7800F scanning electron microscopes (Japan Electronics Co., Ltd., Tokyo, Japan) were used. Energy dispersive spectroscopy (EDS, EDAX lnc., Philadelphia, PA, USA) was applied to analyse the distribution of elements in the weld. Finally, an X-ray diffractometer (XRD) with a Bruker D8-Advance monochromator (Bruker, Karlsruhe, Germany) was used to analyse the phase and content of the weld. In addition, nano-measurer software (Department of chemistry, Fudan University, Shanghai, China) was used to quantify the grain and pore sizes. A microhardness test was carried out using a Huayin HV-1000A hardness tester (Laizhou Huayin Test Instrument Co., Ltd., Yantai, China). The testing load was set to 1 kgf with a holding time of 10 s.

### 2.4. Tensile Properties Test

**Figure 2:**
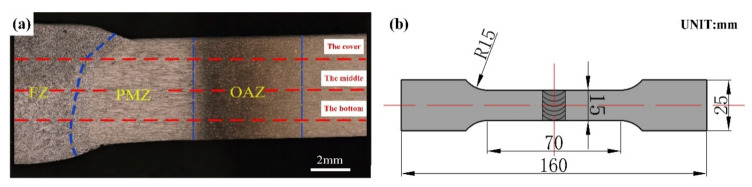
Position of hardness test (**a**) and dimensions of tensile specimen (**b**).

The plate samples were tensile tested using a DDL-300 electronic universal testing machine (Changchun new testing machine Co., Ltd., Changchun, China) with a drawing speed of 1 mm/min. According to the GB/T 228.1-2010 [15] and GB/T 2651-2008 [16] standards, the tensile dimensions of the specimens were designed with a gauge length of 70 mm, as shown in Figure 2b. Five tensile specimens were taken from each weld joint plate, while three tensile specimens were taken from each base metal. The tensile strength and elongation in this paper is all average value.

## 3. Results and Discussion

**Figure 3:**
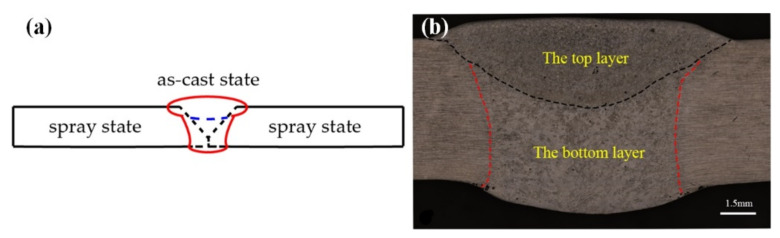
Schematic diagram (**a**) and micromorphology (**b**) of sprayed 2195-T6 Al–Li alloy welded joint.

Figure 3a shows a schematic of the welded joint. The weld was formed into a cast state owing to the melting and recrystallisation. Figure 3b shows the macromorphology of the sprayed 2195-T6 Al–Li alloy joint. As the joining process was divided into two passes, a bottom layer and a top layer can be conducted after welding. The welded joints were divided into a fusion zone (FZ), an EQZ, a PMZ, and an over-aged zone (OAZ), as shown in Figure 2a. The differences in the microstructure between the spray and casting weld joints were analysed.

### 3.1. Microstructures

**Figure 4:**
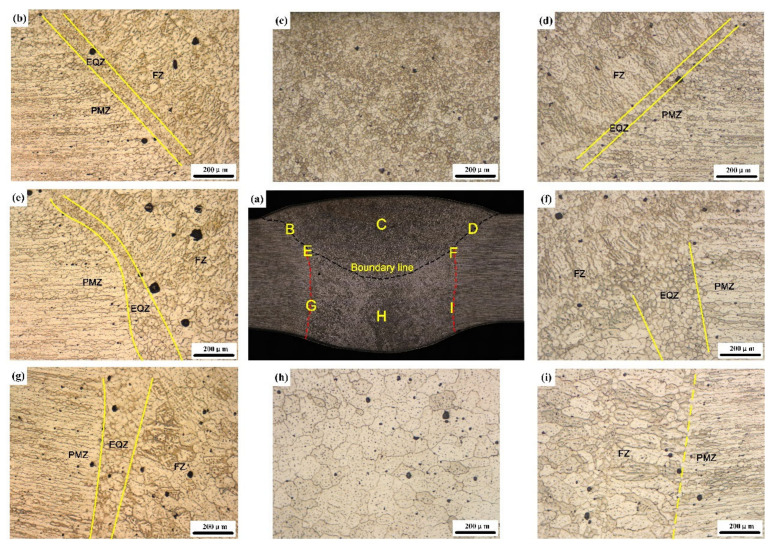
OM images of the (**a**) micro-morphologies of the cross-sections of the sprayed 2195-T6 weld joint: microstructure of the fusion line (**b,d,e**–**g,i**), microstructure of the top layer (**c**) and the bottom layer (**h**).

**Figure 5:**
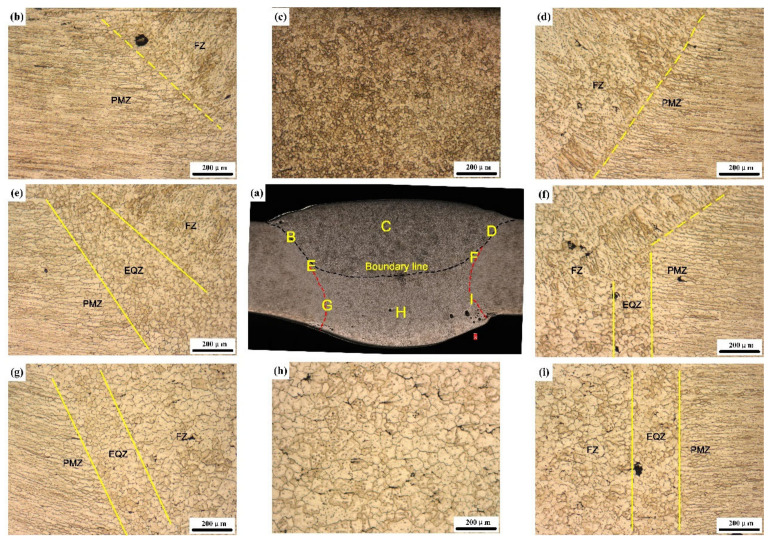
OM image of the (**a**) micro-morphologies of the cross-sections of the cast 2195-T8 weld joint: microstructure of the fusion line (**b,d,e**–**g,i**), microstructure of the top layer (**c**) and the bottom layer (**h**).

**Figure 6:**
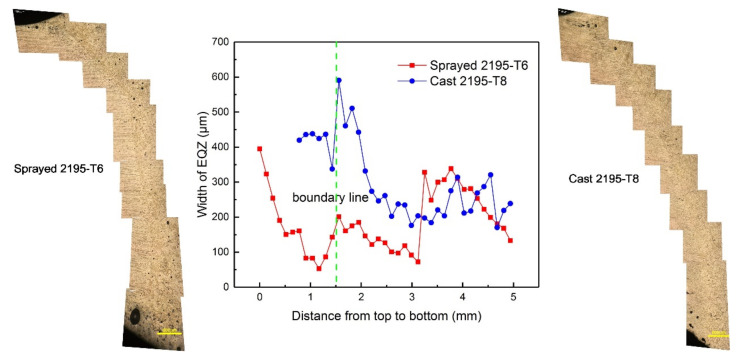
Width distribution of EQZ in sprayed 2195-T6 and cast 2195-T8 aluminium alloy.

This section discussed the microstructure in the weld joint from two aspects: firstly, the formation and growth of weld grain; secondly, the width of EQZ. Figure 4 and Figure 5 show the micro-morphologies of the cross-sections of the sprayed 2195-T6 and cast 2195-T8 aluminium alloy weld joints, respectively. Figure 4b,d,e–g,i shows that the transition zone in the upper, middle, and lower parts of the weld consisted of PMZ and partial EQZ, whereas the EQZ had an average width of 188.7 μm. The width distribution of the EQZ in the joints of the sprayed and cast 2195 alloys are shown in Figure 6. The measurement method is: firstly, according to the scale on the metallographic diagram, determine the corresponding pixel points of the ruler in the photoshop software. Then measure the EQZ width every 0.13 mm from the top to the bottom of the weld joints. It can be seen the width of the EQZ gradually decreased at the upper part and then increased near the boundary line. Furthermore, the width of the EQZ was first reduced and then increased and finally decreased in the bottom layer. Throughout the joint, the FZ adjacent to the EQZ was composed of columnar crystals. The centre of the joint mainly consisted of equiaxed dendrites of various sizes at the top and bottom layers, as shown in Figure 4c,h. As the figures show, the grain size in the bottom layer and approximately 67.80 μm of the joint was larger than that in the top layer approximately 17.82 μm. Figure 4e,f shows that the top layer of the grains increases based on the bottom layer and that the FZ adjacent to the boundary line is also composed of columnar crystals. In the cast 2195-T8 weld joint, the average width of the EQZ was 302.7 μm, and the width distribution tended to be similar to that of the 2195-T6 sprayed weld joint with a large amplitude. The bottom layer did not have columnar crystals adjacent to the EQZ, as shown in Figure 5g,i. In addition, the grain sizes in the bottom layer (approximately 58.45 μm) were smaller than those in the 2195-T6 weld joint, whereas the grain sizes in the top layer (approximately 17.40 μm) were equal. However, there were more cracks in the FZ of the bottom layer and PMZ, as shown in Figure 5f–h. In combination with the crystallisation process, it was inferred that during the solidification process, Cu atoms continuously moved toward the grain boundary; therefore, the freezing point of the grain boundary was reduced, and a liquid film was formed. As the temperature decreased, the other sites were crystallised. However, because no liquid metal supplement was applied in this area, crystal cracks were formed after solidification.

According to the results of Lin et al. [17], an EQZ was mainly formed as a result of the heterogeneous nucleation of Li and Zr. In addition, a low welding parameter and a thin plate can lead to a higher degree of supercooling in front of the solid–liquid interface, which promotes the formation of EQZ [18]. Therefore, the weld joint of the cast 2195-T8 alloy had a wide EQZ.

The columnar crystals grew along some of the equiaxed grains near the fusion line. As a result of the large temperature gradient, the grains easily grew in this region, thus forming columnar crystals. When welding the top layer, a certain temperature of the weld seam remained, and the temperature gradient was relatively smaller than that of the bottom layer; thus, the rate of grain growth was low.

(1) $$\Delta T=D\;G/R\;+A\sqrt{\nu \;\cos\varphi \;/\;\cos\left(\varphi -\theta \right)}$$

Here,

ν = travel speed of the torch;
φ and θ = dendrite alignment angle and grain alignment angle;
A = 2[−2 m (1 − k) C_0_RΓ/D]^1/2^;
D = liquid diffusivity;
m = liquidus slope;
k = partition coefficient;
G = Gibbs–Thompson parameter;
R = grain growth rate.

In addition, the top layer had a fast weld speed; according to (1) [19], the higher the grain growth rate, the lower the undercooling degree and the higher the weld speed, the higher the undercooling degree. Therefore, the supercooling degree (ΔT) was larger at the top layer than at the bottom layer. Consequently, the bottom layer had a larger grain size than the top layer because the bottom layer had experienced twice thermal cycles in welding and the ΔT was smaller.

### 3.2. Al/Cu Element Distribution

#### 3.2.1. XRD Analysis

**Figure 7:**
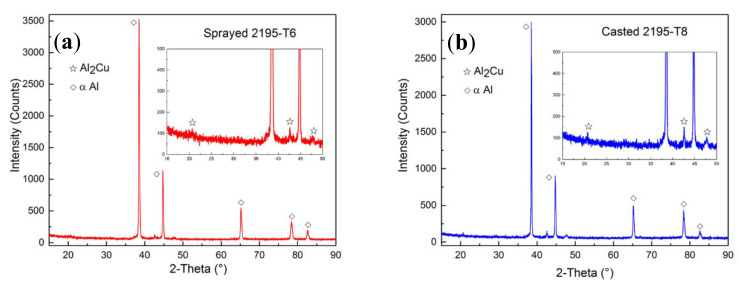
XRD diagrams of weld joints of the sprayed (**a**) and cast (**b**) 2195 aluminium alloy.

Figure 7 shows the XRD analysis results of the phase in the weld joints of the two materials. The 2θ range was between 15° and 90°. It was found that the contained phases were substantially the same in the weld joints regardless of whether sprayed or cast 2195 alloy was applied. In addition, the peak intensities of α-Al were significantly higher than those of the other phases. No other strengthening phases such as T_1_(Al_2_CuLi), δ’(Al_3_Li), or β’(Al_3_Zr) were detected, probably because the base metal has a 90° groove, and the weld joint is mainly composed of filler metal with Cu-rich but Li-poor elements. The Li in the base metal was diluted; thus, the other second phases were smaller and their size was impossible to detect. However, the diffraction intensity of the θ’ phase, namely, Al_2_Cu in the cast 2195 Al–Li alloy, was higher than that of the θ’ phase in the sprayed 2195 Al–Li alloy. This can mainly be attributed to the evident anisotropy of the sprayed 2195-T6 alloy. In Figure 7, the diffraction peak intensity of the aluminium matrix was extremely high, and the second phase was relatively difficult to find. As a result, the diffraction intensity of the θ’ phase in the sprayed 2195 Al–Li alloy was small.

According to the XRD analysis results, the Cu atom clustering was the second phase formed during solidification after welding.

#### 3.2.2. Element Analysis

**Figure 8:**
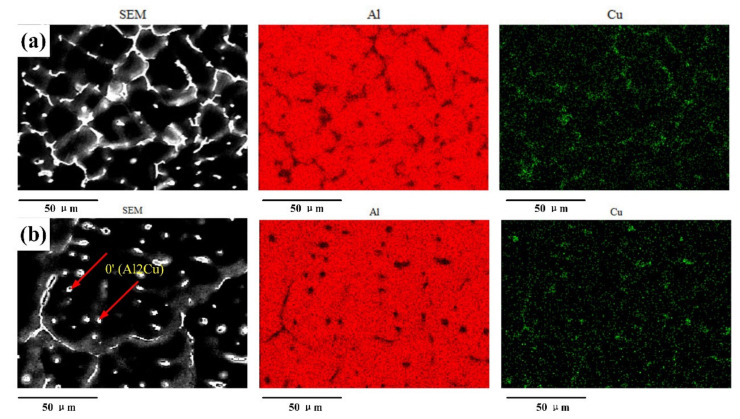
Element mapping in the (**a**) top and (**b**) bottom layers of sprayed 2195-T6.

**Figure 9:**
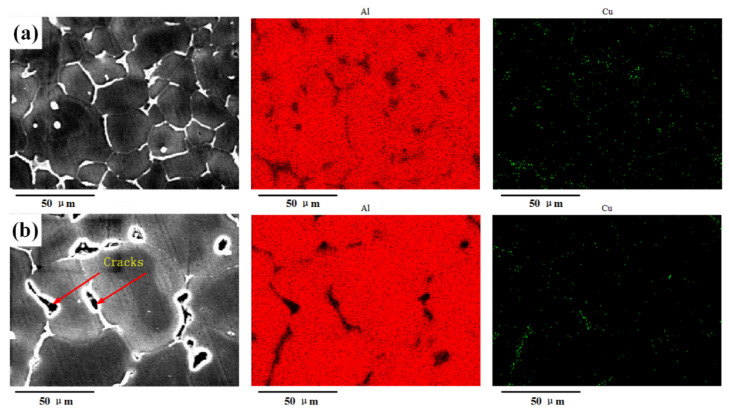
Element mapping in the (**a**) top and (**b**) bottom layers of cast 2195-T8.

Based on the above analysis, a large difference existed in the grain size between the top and bottom layers; the grain size was fine in the top layer and large in the bottom layer. The width of the EQZ differed in the sprayed 2195-T6 and cast 2195-T8 alloys; that is, the width was small in the sprayed 2195-T6 and large in the cast 2195-T8. In addition, the distributions of the Cu and Al elements on the top and bottom layers were studied using EDS. Figure 8 and Figure 9 show the Cu and Al element distributions of the sprayed 2195-T6 aluminium alloy and the cast 2195-T8 aluminium alloy, respectively. It was found that a brighter colour emerged in the FZ of the weld joints, and Cu was mainly distributed at the grain boundary and among the grains. However, small areas of bright colour were found in the cast 2195-T8 alloy. 

**Figure 10:**
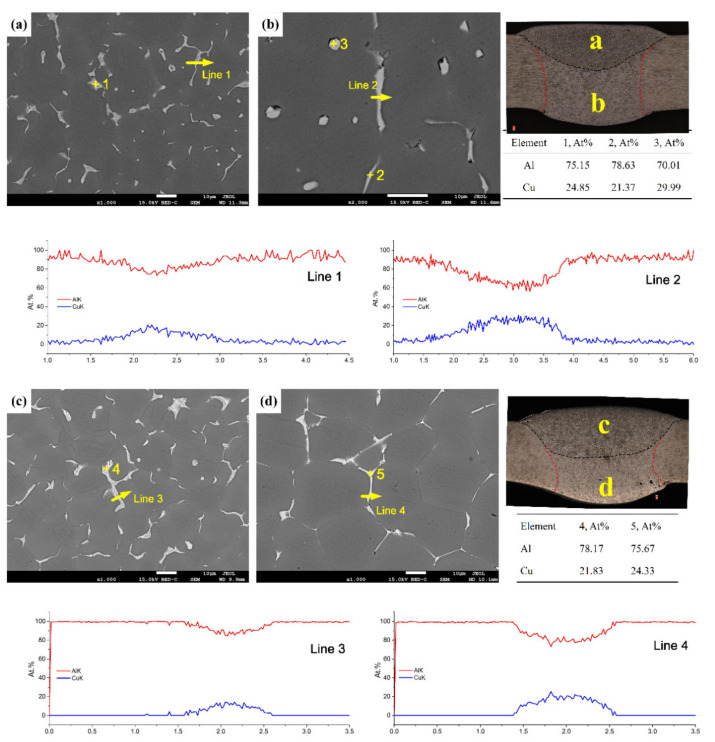
EDS analysis results of (**a**,**b**) sprayed and (**c**,**d**) cast 2195 Al–Li alloys: (**a**,**c**) the top, (**b**,**d**) bottom layers and the Al/Cu element distribution of grain boundaries in lines 1–4.

Figure 10 shows Back scattered Electron (BSE) images and the EDS analysis results of the sprayed and cast 2195 Al–Li alloys. The Al–Cu binary phase diagram, XRD, and EDS analysis results suggest that a brittle eutectic phase (α-Al + θ’) occurred at the grain boundary, whereas the phase of intra-grain precipitation, as shown in Figure 10b, was θ’. The intra-grain precipitation phase of θ’ in the 2195-T6 alloy weld joint was greater than that of the cast 2195-T8 alloy weld joint, as shown in Figure 10b,d. The phase of θ’ was uniformly distributed in the bottom layer, which can lead to dispersion strengthening in the sprayed 2195-T8 alloy weld joint. In addition, the line scanning of the grain boundaries in the top and bottom layers of the FZ in the weld joints indicated that the segregation of Cu elements in the bottom layer was more serious than that in the top layer. Furthermore, the amount of Cu at the grain boundaries was lower in the cast 2195-T8 aluminium alloy weld joint. There were almost no Cu elements adjacent to the grain boundaries in the cast 2195-T8 alloy weld joint, as shown in Figure 10 (Lines 3 and 4), whereas this differed from the weld joint of the sprayed 2195-T6 alloy, as shown in Figure 10 (Lines 1 and 2). Because the welding parameter and the thickness of the plate were slightly smaller in the cast 2195-T8 alloy, the maximum temperature was smaller and the cooling speed was faster than that in the sprayed 2195-T6 alloy. As a result, the diffusion velocity and solid solubility of Cu in the FZ of the cast 2195-T8 alloy weld joint were small, and the amount of Cu was not as high as that of the 2195-T8 alloy weld joint. Therefore, the front edge of the solid–liquid interface showed a large degree of supercooling in its composition, which also promoted the formation of equiaxed crystals and reduced columnar crystals, as shown in Figure 5g,i.

### 3.3. Microhardness Analysis of Weld Joints

**Figure 11:**
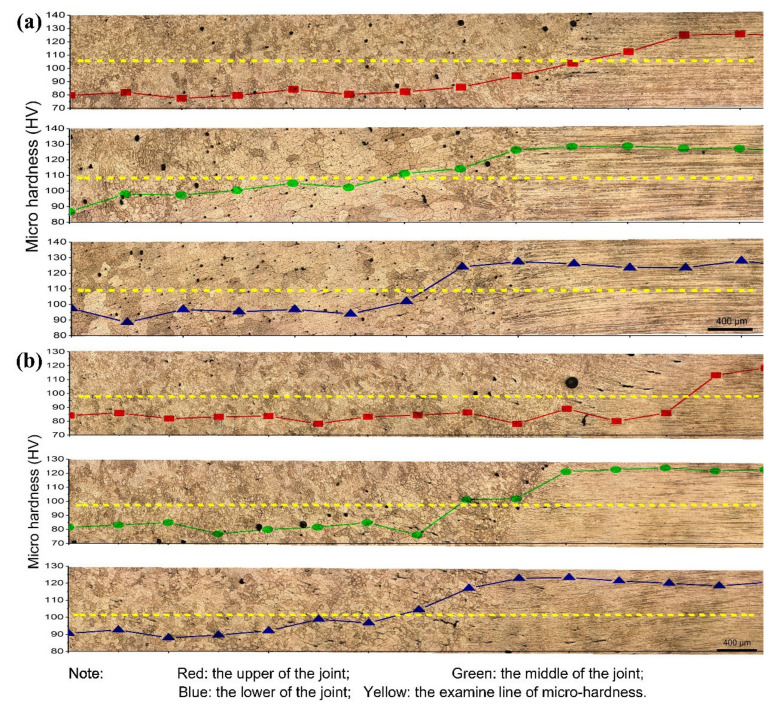
Local microhardness of weld joints near the fusion line: (**a**) sprayed 2195-T6 and (**b**) cast 2195-T8 alloys.

Figure 11 shows the local microhardness of the weld-sprayed 2195-T6 and weld-cast 2195-T8 Al–Li alloy joints. It was found that the microhardness began to increase near the fusion line and then remained constant in both joints. The growth sequence of grains from the centre of the weld to the fusion line of the joint was equiaxed crystal, columnar dendrite, and some fine equiaxed crystal. The microhardness of the equiaxed crystal in the FZ had the lowest value and then slightly increased in the columnar dendrite. In addition, the microhardness of the EQZ was much higher than that of the equiaxed crystal and columnar dendrite in the FZ but not in the PMZ. However, owing to the addition of the Cu-rich filler material in the weld, the main strengthening phase varies from T_1_ in BM to θ’(Al_2_Cu) in the weld seam. Furthermore, because the FZ had not undergone any mechanical formation or heat treatment, the solid solubility of the Cu elements was poor, and, consequently, the microhardness was much lower in the FZ. In addition, more defects were found in the EQZ and PMZ in the weld-cast 2195-T8 alloy joint, as shown in Figure 11b, such as pores and cracks. Lin et al. [17] pointed out that the EQZ in a band-like distribution was prone to cracking when subjected to external forces. Because more cracks were present owing to the nonuniform distribution of microhardness between the EQZ and PMZ in the cast 2195 alloy, damage was likely to occur at this location.

**Figure 12:**
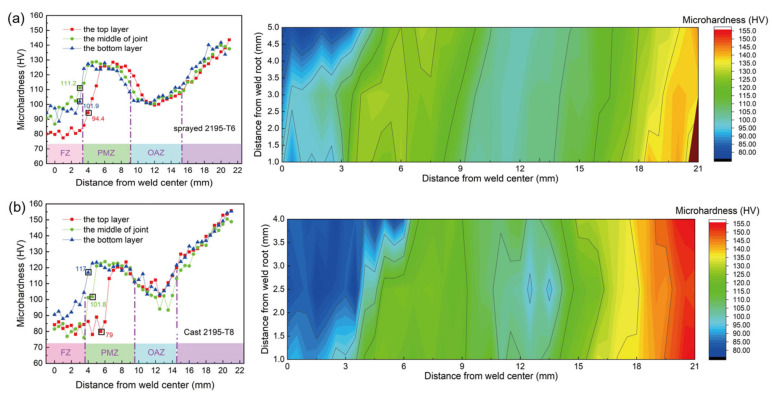
Microhardness distribution of the weld-sprayed 2195-T6 (**a**) and weld-cast 2195-T8 (**b**) aluminium alloy.

Figure 12 shows the microhardness distributions of the weld-sprayed 2195-T6 and weld-cast 2195-T8 aluminium alloy joints. It was found that the microhardness of the top layer had a lower value than that of the bottom layer, which resulted from more θ’ phase particles and an α-Al + θ’ eutectic phase at the bottom layer. 

The hardness of both the EQZ and PMZ increased significantly in the FZ region, as shown in Figure 12, which may have been a result of the fine equiaxed grain size and precipitation, such as in Al_3_Li and Al_3_Zr [17]. It was also found that the microhardness of the bottom layer of the sprayed 2195 weld was higher (approximately 10 Vickers hardness) than that of the bottom layer of the cast 2195, which also confirmed that the degree of element diffusion in the sprayed 2195 weld and the θ’ strengthening phase was higher. The average microhardness of the sprayed 2195 was approximately 5 HV higher than that of the cast 2195 Al–Li alloy on the PMZ. When the cast 2195 alloy transitions from FZ to PMZ, the top and bottom layers differ, and the transition is not smooth. This was mainly attributed to the higher width of the EQZ. Finally, the decreased microhardness of the OAZ is attributed to the melting of the strengthening phases.

### 3.4. Tensile Properties

#### 3.4.1. Tensile Strength and Elongation of the Weld Joints

**Figure 13:**
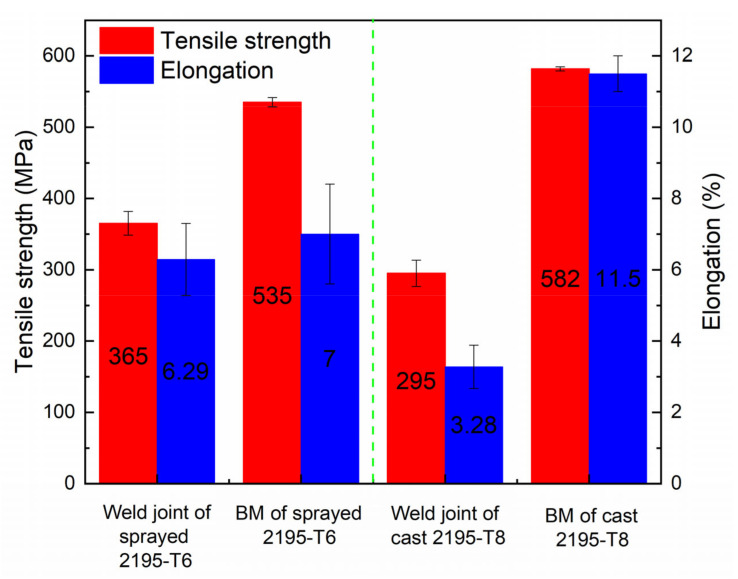
Comparison of the tensile properties between the weld materials of sprayed 2195-T6 and cast 2195-T8.

Figure 13 shows a comparison of the average tensile strength and elongation between the weld materials of the sprayed 2195-T6 and the cast 2195-T8. It was found that the weld-sprayed 2195-T6 aluminium alloy had higher tensile strength and elongation than the weld-cast 2195-T8. The average tensile strength and elongation of the weld-sprayed 2195-T6 were 365 MPa and 6.29%, respectively, which are approximately 68.2% and 89.7% those of the base metal. The average tensile strength and elongation of the weld-cast 2195-T8 were 295 MPa and 3.28%, respectively, which are approximately 50.7% and 28.3% those of the base metal. 

#### 3.4.2. Fracture Morphology of Weld Joints

**Figure 14:**
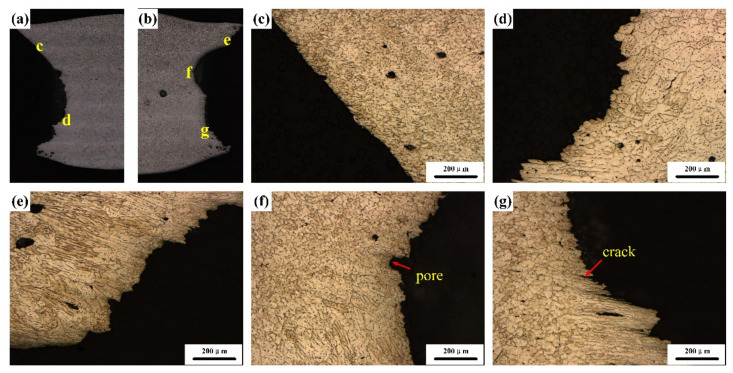
Fracture microstructures of (**a**–**d**) sprayed 2195-T6 and (**e**–**g**) cast 2195-T8 Al–Li alloy weld joints.

**Figure 15:**
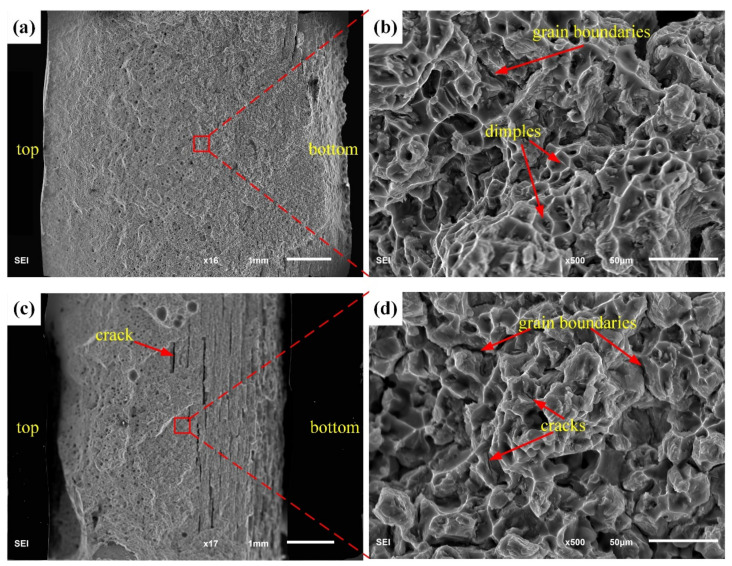
Fracture morphology of sprayed (**a**,**b**) 2195-T6 and (**c**,**d**) cast 2195-T8 Al–Li alloy weld joints.

Figure 14 shows the fracture microstructures of sprayed 2195-T6 and cast 2195-T8 Al–Li alloy weld joints. The fracture position was mainly concentrated within the EQZ and PMZ regions, and some pores and cracks were observed at the fracture section of the cast 2195-T8 Al–Li alloy. Figure 15 shows the morphology of the tensile fractures, where Figure 15a,b represents the fracture morphology of the sprayed 2195 Al–Li alloy and Figure 15c,d represents the fracture morphology of the cast 2195 Al–Li alloy. When the fracture was observed under low magnification, more pores were found in the middle and top layers of the specimen, as shown in Figure 15a,c. In addition, more cracks were found at the bottom layer in Figure 15c. The fracture morphologies in Figure 15b,d are partially magnified from Figure 15a,c. Dimples were homogeneously distributed, but shallow and cracks were present at the grain boundaries shown in Figure 15b. Therefore, it can be concluded that the fracture form of the sprayed 2195-T6 aluminium alloy of a TIG-welded joint is a mixed fracture mode dominated by a plastic fracture. The dimples on the surface of the fracture significantly improved the plasticity of the alloy and effectively improved the overall performance of the material. As shown in Figure 15d, the cracks were present in large amounts at the grain boundaries and among the grains. These pores and cracks proliferate and expand along the grain boundaries when subjected to external forces. In addition, there were almost no dimples in the figure, the surface of the fracture was smooth, and the plasticity of the material obtained by the tensile test was extremely low. Therefore, it was inferred that the cast 2195-T8 Al–Li alloy TIG-welded joint has a mixed fracture mode dominated by a brittle fracture. The distribution of Cu elements near the grain boundaries was too small, resulting in a decrease in the solid–solution strengthening. Furthermore, the second phase strengthening inside the grain was smaller, and the precipitation strengthening was also reduced, thereby decreasing the strength. However, because there are more pores and cracks in the EQZ and PMZ, fractures are prone to occur.

## 4. Conclusions

In this study, the microstructure and mechanical properties of TIG welding joints using sprayed 2195-T6 and cast 2195-T8 Al–Li alloys were studied. The following conclusions can be drawn:

1. The width of the EQZ in a TIG weld joint with cast 2195-T8 Al–Li alloy was approximately 1.6 times larger than the EQZ width found in a weld joint sprayed with 2195-T6 Al–Li alloy. The amount of the second phase θ’(Al_2_Cu) was greater in the weld joint of the cast 2195-T8 Al–Li alloy than that of the sprayed 2195-T6 Al–Li alloy.
2. Cu elements in the weld were mainly distributed at the boundary of the cast 2195 aluminium alloy, whereas the Cu elements were mainly distributed among the grains and at the grain boundaries in the sprayed 2195-T6 Al–Li alloy, which increased the precipitation strengthening effect.
3. The microhardness was approximately 10 HV higher in the bottom layer than in the top layer. In addition, the microhardness was about 10 HV higher in the FZ and PMZ of the joint with sprayed 2195-T6 Al–Li alloy.
4. The fracture positions are all in the EQZ and PMZ. The tensile strength and elongation of the weld joint sprayed with 2195-T6 Al–Li alloy were approximately 68.2% and 89.7% that of the base metal, respectively. However, the tensile strength and elongation of the weld joint with cast 2195-T8 Al–Li alloy were approximately 50.7% and 28.3% that of the base metal.
5. The joint using sprayed 2195-T6 Al–Li alloy showed a mixed fracture mode dominated by a plastic fracture with more dimples. However, the joint using cast 2195-T8 Al–Li alloy showed a mixed fracture mode dominated by a brittle fracture with more pores and cracks.

## Figures and Tables

**Table 1:** Chemical compositions of base metal and wire (wt %).

Materials	Cu	Li	Mg	Ag	Zr	Fe	Mn	Si	Cr	Ti	Zn	Al
Sprayed 2195-T6	3.66	0.88	0.45	0.31	0.12	0.036	0.0006	0.067	-	-	-	Bal.
Cast 2195-T8	4.02	1.0	0.4	0.41	0.11	0.16	-	0.03	-	-	-	Bal.
Filler wire	5.6	-	-	-	0.095	0.378	0.327	-	0.084	-	-	Bal.
A92195	3.7–4.3	0.8–1.2	0.25–0.8	0.25–0.6	0.08–0.16	≤0.15	≤0.25	≤0.12	-	≤0.1	≤0.25	Bal.

**Table 2:** Mechanical properties of sprayed 2195-T6 and cast 2195-T8 Al–Li alloys.

Materials	Ultimate Tensile Strength (MPa)	Elongation (%)
Sprayed 2195-T6	535	7.0
Cast 2195-T8	590	11.6

**Table 3:** Welding parameters of sprayed 2195-T6 and cast 2195-T8 aluminium alloys.

Materials	I_bottom_ (A)	U_bottom_ (V)	V_bottom_ (mm/min)	Heat Input (KJ/cm)	I_top_ (A)	U_top_ (V)	V_bottom_ (mm/min)	Heat Input (KJ/cm)
Sprayed 2195-T6	198	14.5	115.8	14.9	173	16.0	126.6	13.1
Cast 2195-T8	185	14.4	126	12.7	169	15.6	135	11.7
